# Early Cell Adhesion of Periodontal Ligament Stem Cells on 3D Printed Polylactic Acid/Hydroxyapatite Scaffolds: An In Vitro Study

**DOI:** 10.3390/polym17223088

**Published:** 2025-11-20

**Authors:** Ildefonso Serrano-Belmonte, Javier Montero, Elena Guerrero-González, Alexandra Munteanu, Virginia Pérez-Fernández, Amparo Pérez-Silva, Ascensión Martínez-Cánovas

**Affiliations:** 1 Faculty of Medicine, School of Dentistry, Clínica Odontológica Universitaria, 2 Planta, Hospital Morales Meseguer, University of Murcia, Avda. Marqués de los Vélez s/n, 30008 Murcia, Spain; elena.g.g@um.es (E.G.-G.); ai.munteanu@um.es (A.M.); amparo.perez@um.es (A.P.-S.); ascension.martinez4@um.es (A.M.-C.); 2 Department of Surgery, Faculty of Medicine, University of Salamanca, 37008 Salamanca, Spain; javimont@usal.es; 3 Department of Social and Health Sciences, Institute of Biomedical Research (IMIB-Arrixaca), University of Murcia, 30120 Murcia, Spain; virperez@um.es

**Keywords:** polylactic acid, bone regeneration, scaffolds, biocompatibility, cell adhesion, hydroxyapatite, additive manufacturing

## Abstract

Polylactic acid (PLA) and its composites with hydroxyapatite (HA) have been studied in the field of bone repair applications. The objective of this study was to evaluate the biocompatibility of PLA/HA at different concentrations and to analyze early adhesion of periodontal ligament stem cells (PDLSCs). Cells were seeded in two 24-well plates, each containing six disk-shaped samples of PLA/HA (10%, 15% and 20%) and six control samples and then examined using scanning electron microscopy. Twelve 96-well plates were prepared with different elution concentrations (1/1, 1/2, 1/4, and 1/8) to assess biocompatibility using MTT cell viability and Hoechst 33342 assays at 24, 48, and 72 h. PLA/HA 20% showed the highest early adhesion (*p* = 0.0057), with cells adopting a more elongated morphology. The MTT assay revealed no differences in viability between concentrations (*p* = 0.6196), whereas the Hoechst assay demonstrated the highest viability for PLA/HA 20% (*p* < 0.0001). Overall, PLA promoted cell adhesion, with the 20% formulation providing the greatest adhesion. All concentrations maintained high viability, and longer culture time enhanced both adhesion and viability.

## 1. Introduction

**Figure 1:**
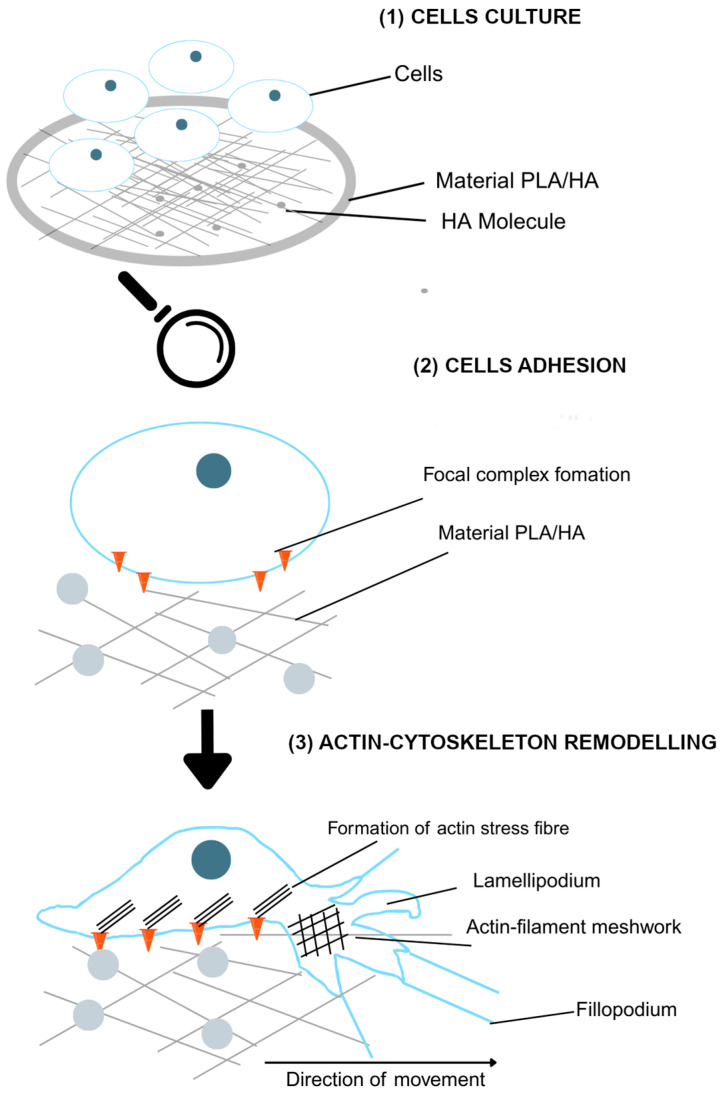
Dynamic diagram of the culture, adhesion, and morphological changes that occur in PDLSCs upon contact with PLA/HA discs. Looking at the magnification, the cells present complexes that, upon detecting the surface, may allow HA molecules to enhance this complex formation, leading to the generation of actin fibers that favor the creation of fillopodium and lamellipodum which promote cell extension, adhesion, and migration, seeking to spread and contact other cells.

Following tooth extraction, physiological resorption occurs in the underlying socket, which can result in loss of support for future prosthetic rehabilitation. To address this, strategies are being investigated to enhance healing and prevent bone loss, with tissue engineering as a promising approach. Scaffolds, used as structural niches, aim to recreate the three-dimensional cellular matrix and provide topological and physicochemical cues to cells [1]. In essence, they serve as a temporary framework that supports the attachment, proliferation, and differentiation of progenitor cells [2]. These scaffolds must be biocompatible, biodegradable, and mechanically suitable to allow effective cell adhesion. Various alloplastic grafts are under investigation, with additional requirements including low cost, stability during the time needed for new bone formation, and complete resorption within 6 to 12 months [3]. Among these, polylactic acid (PLA) is a synthetic polymer notable for being bioabsorbable, biodegradable, biocompatible, and immunologically inert. However, PLA has limited mechanical strength, which has prompted its combination with other materials such as hydroxyapatite (HA). HA, a bioactive ceramic and a major component of bone and teeth, is characterized by its porosity, resorbability, and absence of toxic metabolites. It does not elicit a foreign body reaction and is highly biocompatible and osteoconductive [4]. Combining PLA with HA not only enhances scaffold strength but also produces a composite more structurally and chemically similar to bone tissue. This combination improves osteoconductive, osteoinductive, and osteogenic properties while slowing degradation by buffering acidic by-products [5,6,7]. However, PLA/HA composites present challenges, including potential HA agglomeration within the PLA matrix and weak bonding between hydrophobic PLA and hydrophilic HA, which may compromise mechanical properties. Adjusting HA concentration is one strategy to improve dispersion and compatibility with the polymer matrix [8] (Figure 1).

Among the various qualities required, the primary criterion in tissue matrix engineering is biocompatibility, which refers to the ability of a material to permit cell adhesion, migration, and proliferation without eliciting an adverse immune response or severe inflammation [9,10]. Accordingly, it is essential to study the behavior of cells that will interact with these systems. Although osteoblast induction is critical for bone formation, the cells of the periodontal ligament must also be considered to ensure proper healing and consolidation of regenerated tissue.

Periodontal ligament stem cells (PDLSCs) are unspecialized cells with the capacity for self-renewal, multipotency, and immunomodulation. They can differentiate into mesenchymal lineages, giving rise to osteoblasts, chondrocytes, and adipocytes [11], which justifies their use in this study.

The objective of the present research was to evaluate the response of PDLSCs when exposed to PLA/HA composites at different concentrations, focusing on cell proliferation and adhesion, as well as potential biocompatibility and cytotoxicity over time.

## 2. Materials and Methods

### 2.1. Obtaining the Material

PLA combined with HA at concentrations of 10%, 15%, and 20% by volume was used. The material was supplied by Colfeed (Madrid, Spain) and Regemat3D, S.L. (Granada, Spain) in filament format and divided into two groups. The first group was processed at the Research Support Workshops of the Scientific and Technical Research Area (ACTI) of the University of Murcia using a Creality Ender 3 V3 SE 3D printer (Shenzhen, China). The filament diameter was 1.75 mm (±0.15 mm), and the printing temperature ranged from 155–165 °C, producing disks 2 mm thick and 10 mm in diameter. The second group consisted of untreated filaments, which were cut into irregular fragments for elution.

### 2.2. Sample Preparation

Both disk-shaped and filament-shaped samples were sterilized prior to use. They were immersed in pure ethanol, rinsed with distilled water, and dried to remove bacteria, impurities, and grease residues. Sterilization was then completed in a UV-C laminar flow cabinet at 254 nm for 30 min on each side. The prepared material was diluted in 20 mL of DMEM culture medium without phenol red and incubated for 24 h at 37 °C in 7.5% CO_2_ and 85% relative humidity.

### 2.3. Cell Culture

To carry out the study, the PDLSCs, previously stored in liquid nitrogen at –196 °C in the Cell Culture Service of the University of Murcia, were thawed. For the maintenance and conservation of the cells throughout the study, a culture medium was prepared containing Dulbecco’s Modified Eagle Medium (DMEM) with phenol red, 10% fetal bovine serum (FBS), 4.5 g/L glucose, 4 mM glutamine, and 1% penicillin. Once the medium was prepared and the cells were thawed, they were added to a centrifuge tube with 9 mL of culture medium. They were then centrifuged at 300× *g* for 7 min, the supernatant was removed, and 2 mL of fresh culture medium was added. Finally, 100 μL of this mixture and 100 μL of trypan blue were mixed in a 0.5 mL tube to determine the total number of cells and to assess viability. After counting with the TC20™ Automatic Cell Counter, 100% viability was obtained with a total of 5.22 × 10^5^ cells/mL, corresponding to a seeding density of 10,000 cells/cm^2^. The first cell culture was carried out by incubating the cells at 37 °C, 7.5% CO_2_, and 85% relative humidity. Subsequently, three subcultures were performed when the cells occupied approximately 85% of the flask surface to ensure survival and avoid excessive confluence and nutrient depletion.

### 2.4. Cell Seeding

#### 2.4.1. Seeding of Wells for the Study of Adhesion and Cell Morphology

Two 24-well polystyrene culture plates for suspended cells (Sarstedt Inc., Newton, MA, USA) were used, one evaluated at 48 h and the other at 72 h, with a seeding density of 25,000 cells per well. The disks were arranged as follows: in the first row of six wells, 10% PLA/HA disks were placed (one per well); in the second row, 15% PLA/HA disks; in the third row, 20% PLA/HA disks; and in the fourth row, sterile 13 mm plastic coverslip control disks designed for tissue culture and surface-treated for cell adhesion (Sarstedt Inc., Newton, MA, USA). The plates were then returned to the incubator under the same conditions described above.

#### 2.4.2. Seeding of Wells for Viability and Apoptosis Studies

Cell seeding was performed using twelve 96-well plates. PLA/HA 10% and PLA/HA 15% samples shared plates, while PLA/HA 20% samples were placed separately. Of the twelve plates, six were used for the MTT assay at 24, 48, and 72 h, and six for the Hoechst assay at the same points.

Each plate included water-filled wells along the edges, positive control wells (n = 6), wells for different elutions (n = 6 for each elution), negative control wells (n = 6, where cell death was included with DMSO), and blank wells (n = 2, without cells). For each sample type (10%, 15% and 20%), wells were allocated for the four elutions: n = 6 wells each for 1/1, 1/2, 1/4, and 1/8 elution. Each well with cells had a seeding density of 7500 cells/well.

### 2.5. Sample Processing

#### 2.5.1. Adhesion and Cell Morphology Test

After 48 h, the culture medium was removed from each well. Cells were fixed with 2.5% glutaraldehyde (1 mL per well) for 25 min. The fixative was then removed, and 1 mL of wash solution (0.1 M cacodylate buffer with sucrose) was added to each well. The samples were subsequently dehydrated in acetone with increasing concentrations and dried at the critical point. Once dried, they were coated with a 5 nm layer of platinum.

After 72 h, the same procedure was repeated for the corresponding plate.

After platinum coating, images were obtained using an ApreoS Field Emission Scanning Electron Microscope (FESEM) (Thermo Scientific, Brno, Czech Republic). For each of the 24 samples, five images at 300× magnification were taken to analyze the cell-covered surface area, and one image at 1200× magnification was taken to evaluate cell morphology.

#### 2.5.2. Cell Viability Assay (MTT)

The MTT assay was performed at 24, 48, and 72 h. First, the medium was removed from the negative control wells, and 100 μL of DMSO was added for 5 min and then discarded. The medium was removed from the remaining wells, and 200 μL of MTT solution (1 mg/mL in DMEM without phenol red and without SBF) was added to each well under dark conditions. The plates were wrapped with aluminum foil to prevent light exposure and incubated at 37 °C, 7.5% CO_2_, and 85% relative humidity for 4 h. After incubation, the MTT solution was removed, and 100 μL of DMSO was added. Plates were shaken for 5 min at 200 rpm, and absorbance was measured using a FLUOstar Omega plate reader (BMG LABTECH, Ortenberg, Germany) at a wavelength between 520 and 580 nm.

#### 2.5.3. Apoptosis or Cell Death Assay

To evaluate cell apoptosis, Hoechst 33342 dye was used. For each assay day and for each plate, the following protocol was applied. The medium from the negative control wells was removed, and 100 μL of DMSO was added, left to act for 5 min, and then removed. From the sample wells, 50 μL of culture medium was removed and replaced with 100 μL of Carnoy’s fixative solution (3:1 ratio of absolute methanol and glacial acetic acid). The solution was left for 5 min and then removed. Next, 200 μL of Carnoy’s fixative solution was added again to fill the well, and all liquid was removed after 10 min. The plates were then allowed to dry for 1 h. Then, 200 μL of Hoechst solution (1 μg/mL in phosphate-buffered Saline, PBS) was added and left at room temperature in darkness. After incubation, the solution was removed, and the wells were washed three times with distilled water. The plates were allowed to dry in darkness. The plates were analyzed using a Nikon Eclipse TE2000-U inverted microscope (Tokyo, Japan) at a 50 μm scale, and three photographs were taken of each well, ensuring that at least 50 stained nuclei were captured in each.

### 2.6. Image Analysis

The images obtained from each process were analyzed using the free software Fiji 2.17.0. In collaboration with the Image Analysis Service of the University of Murcia, three macros were created. Two were developed for analyzing cell adhesion samples: the Threshold-Yen_v2 Cell Count macro for PLA/HA samples and the Manual Cell Counting macro for control disks. For the apoptosis or cell death assay with Hoechst 33342, the ConteoCelulas-v7 macro was created to determine the number of living and dead cells. In this macro, living cells were identified by rounder shapes, whereas dead cells were identified by more irregular shapes, regardless of whether death occurred by programmed apoptosis or necrosis.

### 2.7. Statistical Analysis

Statistical analyses were performed using Stata V14 (StataCorp LLC). A descriptive analysis of the variables of interest was conducted, reporting mean and standard deviation, median (p50), and interquartile range (p25–p75). Normality of the variables was then assessed.

The Mann–Whitney U Test was applied to analyze the percentage of area occupied by cells as a function of elapsed time. For comparisons involving more than two means, the Kruskal–Wallis test was used, followed by the Dunn-Bonferroni post hoc test for pairwise comparisons. A significance level of α = 0.05 was established.

## 3. Results

### 3.1. Cell Adhesion

**Figure 2:**
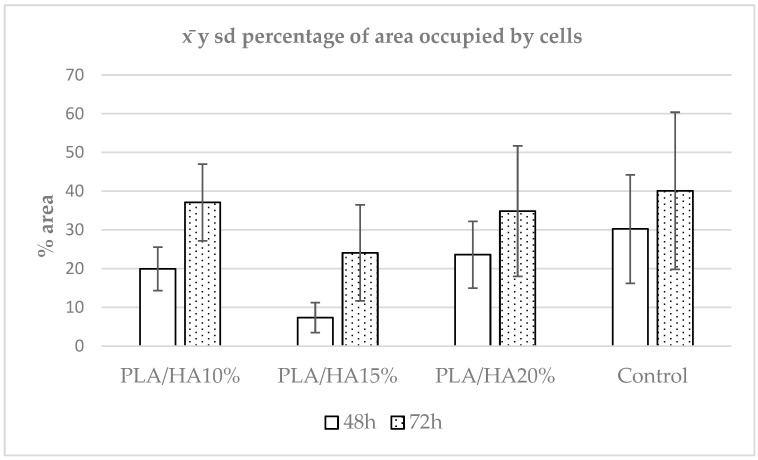
Mean and standard deviation of the percentage of area occupied by cells when compared at different time points.

A descriptive analysis of the samples was performed. The mean percentage of occupied area was 26.74%, with a standard deviation of 16.00297, a median (p50) of 24.94732, an interquartile range of P25 = 15.92287 and P75 = 34.51661, a minimum of 0.64174, and a maximum of 82.91863 (Figure 2).

The Mann–Whitney U test was used to compare results between materials at 48 and 72 h. Significant differences were observed in the PLA/HA 10%, PLA/HA 15%, and PLA/HA 20% groups. However, in the control group, *p* = 0.0948, indicating no significant differences between 48 and 72 h in this group.

Using the Kruskal–Wallis test, the set of materials was compared within the same time interval. At both 48 and 72 h, significant differences were found among the samples. To identify the specific groups with differences, the Dunn-Bonferroni test was applied for pairwise comparisons. At 48 h, significant differences were observed between PLA/HA 10% and PLA/HA 15%, PLA/HA 15% and PLA/HA 20%, PLA/HA 10% and the control group, and PLA/HA 15% and the control group (Table 1).

**Figure 3:**
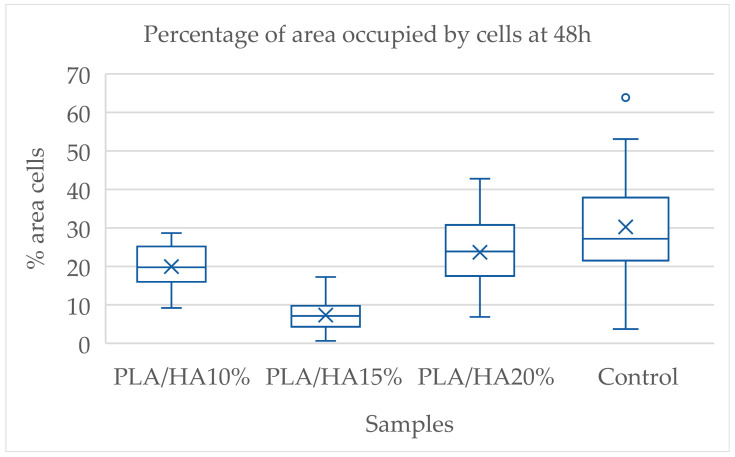
Percentage of area occupied by cells at 48 h.

According to the data, the control group showed the highest value, followed by PLA/HA 20%, PLA/HA 10% and PLA/HA 15%. This suggests that cells adhered more strongly to the material with the highest HA concentration, although the control group still exhibited the highest adhesion overall. The differences were statistically significant compared with all other samples except PLA/HA 20% (Figure 3).

The same comparison was conducted at 72 h. Statistically significant differences were observed between PLA/HA 10% and PLA/HA 15%, PLA/HA 15% and PLA/HA 20% and PLA/HA 15% and the control group (Table 2).

**Figure 4:**
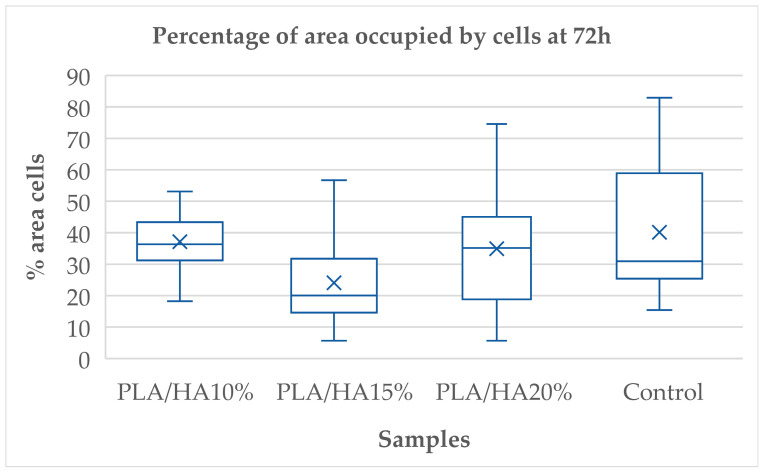
Percentage of area occupied by cells at 72 h.

The control group occupied the highest percentage of area, followed by PLA/HA 20%, PLA/HA 10%, and finally PLA/HA 15%. Among the materials, PLA/HA 20% again showed better results, with no statistically significant differences compared to the control group (Figure 4).

### 3.2. Morphological Analysis

**Figure 5:**
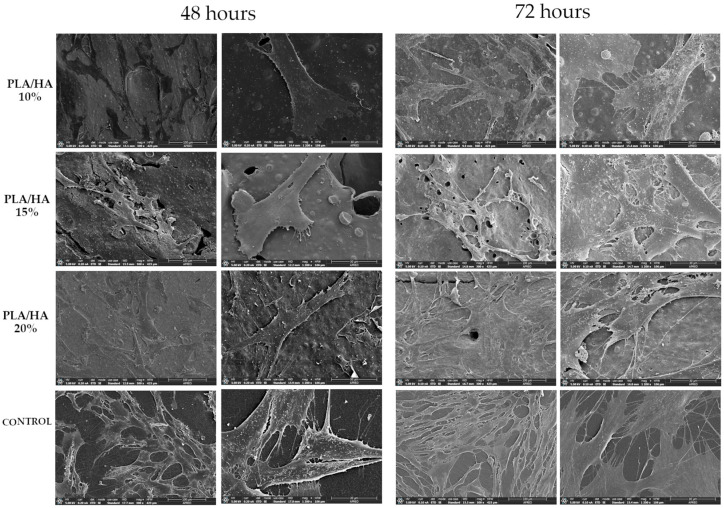
300× and 1200× photographs of each of the materials using FESEM.

PDLSCs initially presented a rounded shape; however, over time and in contact with certain materials, they acquired a more stellate and elongated morphology. They also developed extensions of the cytoplasm, such as filopodia and lamellipodia, that allow them to extend over the surface of the material, in addition to facilitating the connection between cells with each other. Changes in this cell morphology were observed with FESEM at 48 and 72 h (Figure 5).

PLA/HA 10%: At 48 h, cells exhibited a flattened morphology with limited lamellipodia extension. By 72 h, cells began to elongate, with more lamellipodia formation (narrow, not widely extended) and few filopodia.

PLA/HA 15%: Compared with PLA/HA 10%, a higher cell density is observed. Filopodia and short lamellipodia are present. At 72 h, there was a marked increase in cell spreading, with elongated morphology and the presence of filopodia and lamellipodia.

PLA/HA 20%: At 48 h, cell density is higher than in both PLA/HA 10% and 15%. Distinct filopodia and lamellipodia are evident. At 72 h, cells displayed advanced development, with stellate or elongated morphology and multiple extensive cytoplasmic projections.

Control group: At 48 h, high cell density is evident, with an elongated morphology and multiple filopodia and lamellipodia. At 72 h, the surface is nearly covered with elongated cells, showing well-developed filopodia and lamellipodia, indicating adhesion both to the substrate and between adjacent cells.

### 3.3. Cell Viability Assay Using MTT

**Figure 6:**
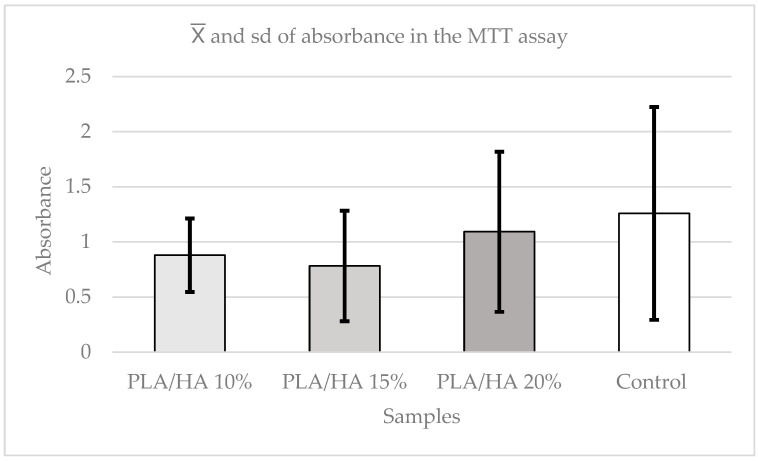
Absorbance graph according to concentrations in the MTT test.

The Kruskal–Wallis test was used to evaluate potential differences among the materials. The analysis showed no statistically significant differences between the control, PLA/HA 10%, PLA/HA 15%, and PLA/HA 20% (*p* = 0.6196). Even so, the control group exhibited higher cell viability, whereas PLA/HA 15% showed the lowest values among the materials (Figure 6).

**Figure 7:**
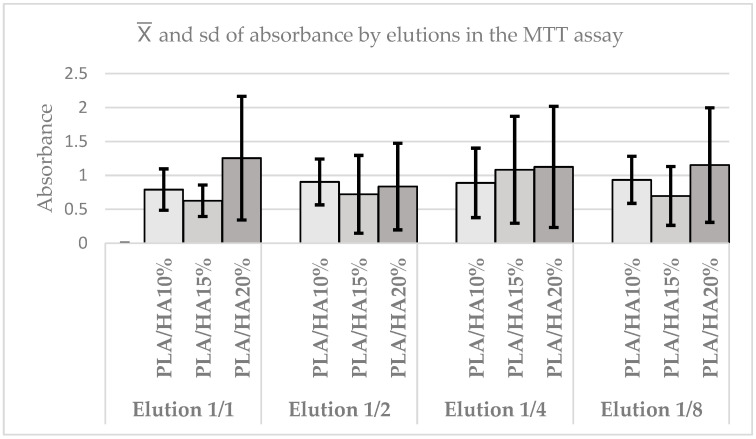
Absorbance graph according to elutions in the MTT test.

In the same way, the absorbance between the different materials by elution was compared. In the case of the 1/1 elution, it was concluded that there were no statistically significant differences between them (*p* = 0.6703). The same occurred in the 1/2 elution (*p* = 0.8752), in the 1/4 elution (*p* = 0.8752), and in the 1/8 elution (*p* = 0.5611). It was observed that although the elution of all materials increases, the differences between them are not enhanced, and in all elutions, no statistically significant differences are found. In any case, the highest cell viability is obtained with 20% PLA/HA, except in 1/2 elution, where 10% PLA/HA predominates. Likewise, it is observed that absorbance tends to be maintained or slightly increased with elutions, especially in PLA/HA 10% and PLA/HA 20% (Figure 7).

**Figure 8:**
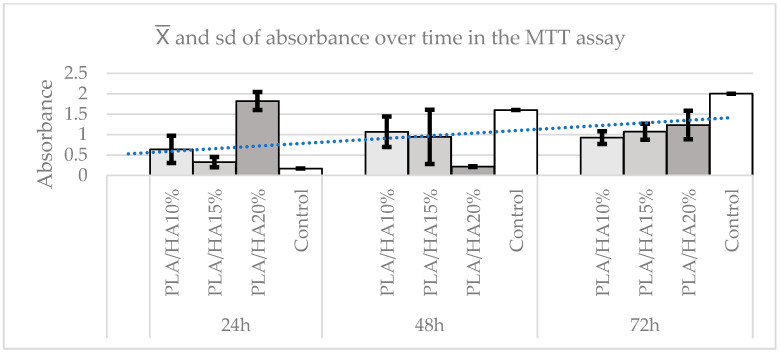
Absorbance graph according to time in the MTT test. It is a line that marks the average and the trend that the sample follows.

Regarding the difference in absorbance between materials over time, the Kruskal–Wallis test was used, finding statistically significant differences between them (*p* = 0.0244). To compare the different pairs, Dunn’s test was applied, which revealed that over time, the control group showed continuous growth, while the other materials showed more variable behavior. At 24 h, there were no statistically significant differences between the materials and the control group (*p* ≥ 0.1373), except for PLA/HA 20%, which presented an increase in absorbance with differences compared with all groups (*p* ≤ 0.05). This effect disappears after 48 h, when the control group showed the highest cell count, with statistically significant differences (*p* = 0.0375) compared with the other materials. Although PLA/HA 10% was the material that performed best, there were no statistically significant differences between the groups (*p* ≥ 0.1945), except between PLA/HA 20% and the other materials (*p* ≤ 0.0184). Finally, at 72 h, no statistically significant differences were found (*p* = 0.2167) between the groups, although the control group continued to show the highest cell viability, followed by PLA/HA 20%, which obtained the best absorbance results (Figure 8).

### 3.4. Apoptosis or Cell Death Assay

**Figure 9:**
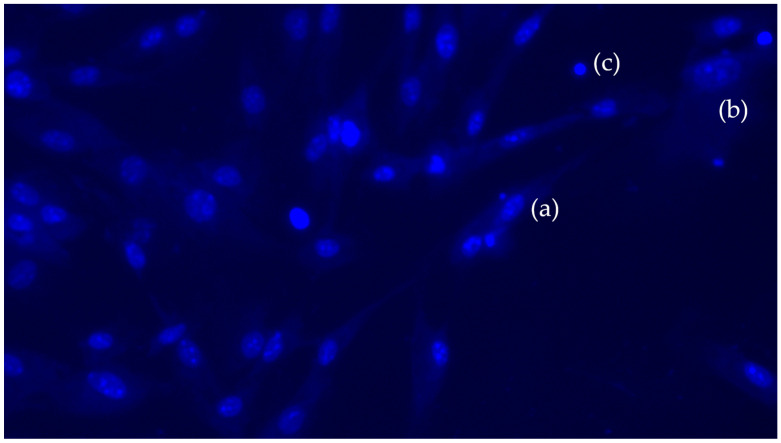
The blue tones shows the presence and intensity of the stained DNA. The more intense the blue, the more DNA or the greater the condensation. Image of living (**a**), the nuclei appear round and evenly staines. In the apoptotic cells (**b**) the nuclei are generally fragmented and staines more intensely because of condensation of the DNA. In necrotic cells (**c**) the DNA is not condensed and the edges of the nucleus are less clearly defined. In healthy, untrated cells, the nuclei appear round and evenly staines.

To evaluate cell apoptosis, Hoechst 33342 was used, which stains cell nuclei blue (Figure 9), regardless of whether the cell is living or dead. This dye was applied to the PLA samples with 10%, 15%, and 20% HA to perform the cell count, as mentioned above, using the Fiji software.

By means of the Kruskal–Wallis test, it was determined that there were differences in cell viability between the materials (*p* = 0.0001). To determine which materials these differences were between, Dunn–s test was used, confirming that there were statistically significant differences between all materials and the control group (*p* ≤ 0.0092), except between PLA/HA 10% and PLA/HA 15% (*p* = 0.2247).

**Figure 10:**
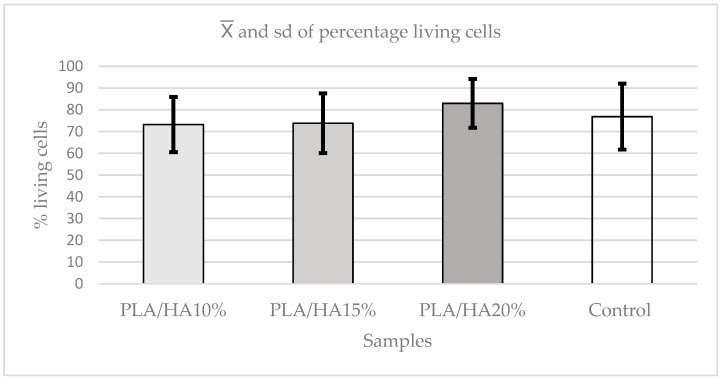
Percentage of cell viability according to concentrations in the Hoechst assay.

The material with the most cell viability among all of them is PLA/HA 20%, while PLA/HA 10% and PLA/HA 15%, which do not differ significantly, obtained the worst cell viability results (Figure 10).

In the 1/1 elution, statistically significant differences were found between the materials (*p* = 0.0001). These occurred between PLA/HA 20% and the other materials (*p* ≤ 0.0001), but not between PLA/HA 10% and PLA/HA 15% (*p* = 0.1244). In the 1/2 elution, statistically significant differences were observed (*p* = 0.0001); again, PLA/HA 20% differed from the other materials (*p* ≤ 0.0001), while no difference was found between PLA/HA 10% and PLA/HA 15% (*p* = 0.1932). In the 1/4 elution, the same pattern of statistically significant differences between the materials was repeated (*p* = 0.0036). Finally, in the 1/8 elution, statistically significant differences were also found between the materials (*p* = 0.0003).

**Figure 11:**
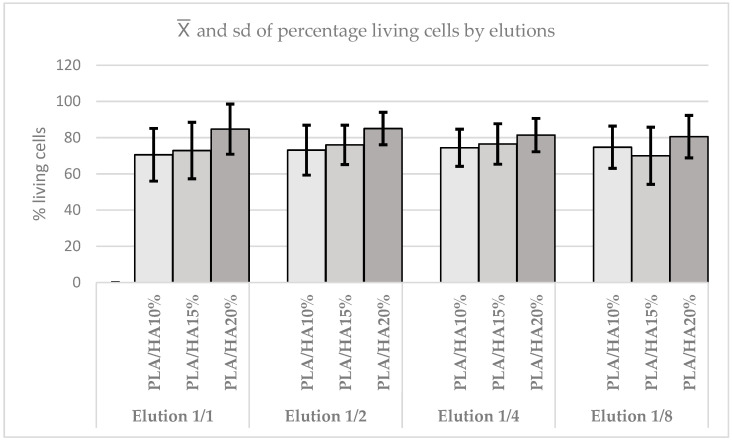
Percentage of cell viability according to elutions in the Hoechst assay.

It was observed that as the elution of all materials increases, statistically significant differences persist, with PLA/HA 20% consistently showing the highest viability (Figure 11).

To observe the differences between materials within each time point, the same statistical method was applied. Significant differences were found at 24, 48, and 72 h.

**Figure 12:**
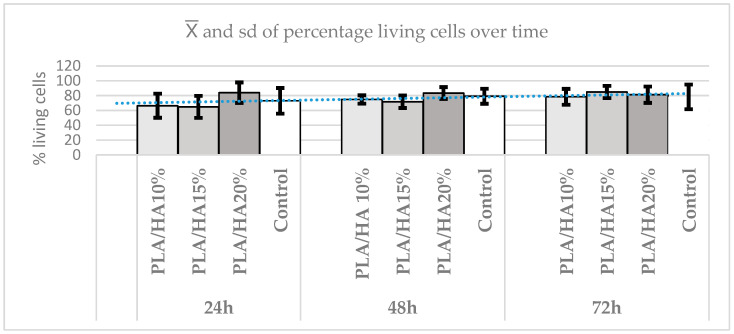
Percentage of cell viability according to time in the Hoechst assay (blue line is the sample mean).

As time progressed, the differences tended to decrease, and cell viability became more similar among the groups. PLA/HA 20% maintained the highest viability over time, except at 72 h, when PLA/HA 15% showed the highest viability (Figure 12).

**Figure 13:**
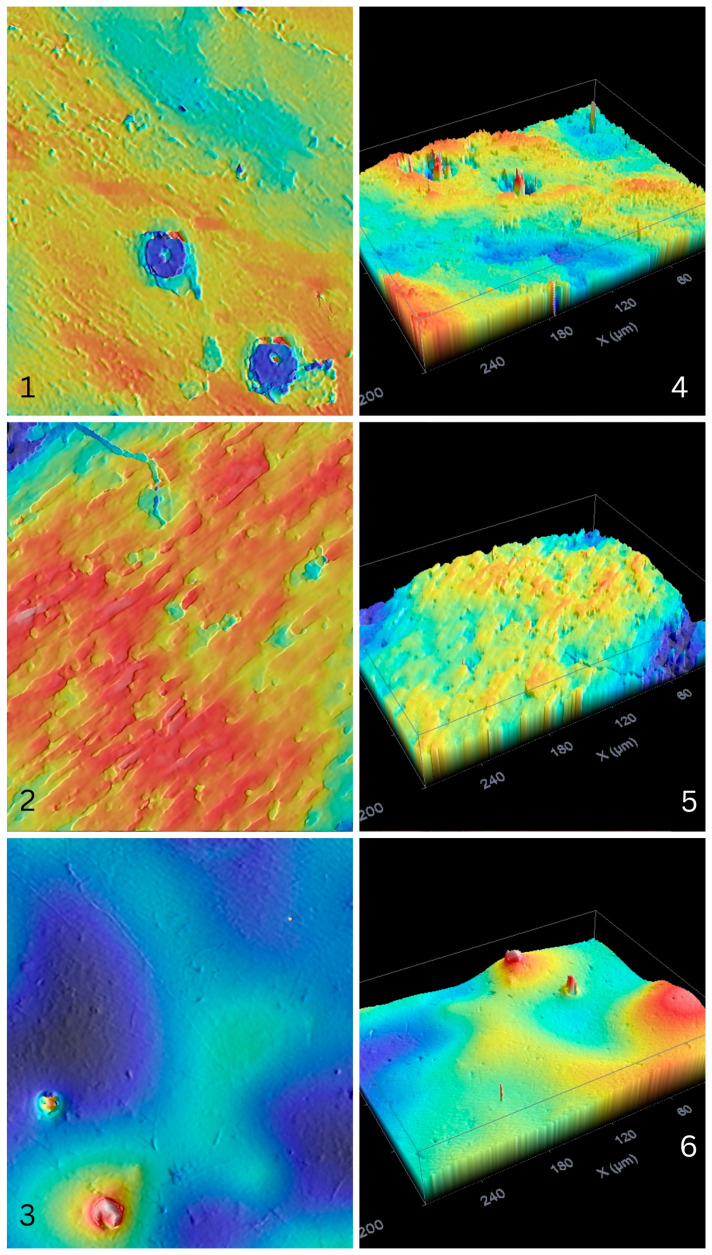
White light microscope micrographs. 1, PLA/HA 10 2D. 2, PLA/HA 15 2D. 3, PLA/HA 20 2D. 4, PLA/HA 10 3D. 5, PLA/HA 15 3D. 6, PLA/HA 20 3D (scan size 294 × 214 µm).

Table 3 shows roughness and wetting data. A significant influence was found in the roughness parameters and contact angle. Wetting was higher in the PLA/HA 20 group. Figure 13 shows 2D and 3D topographies, showing different morphologies.

## 4. Discussion

Various studies have analyzed how different cells (such as PDLSCs, osteoblasts, fibroblasts, mesenchymal stem cells, etc.) adhere to different materials (including PLA, collagen, cellulose, alginate, PLC, polyethylene glycol, etc.) that are used in the manufacture of scaffolds [12]. In the present study, PDLSCs were chosen because studies such as that of Lee et al. [13] and Ge et al. [14] have demonstrated their efficacy for the regeneration of dental and periodontal tissues.

Campos-Bijit et al. [15], Pizzicanella et al. [16] and Neshati et al. [17] used FESEM to analyze cell adhesion and morphology, although with different materials (titanium dental implants, hydroxyapatite scaffold and gelatin/siloxane/HA scaffold respectively) and different cells (retromolar gingival mesenchymal stem cells, PDLSCs and mesenchymal stem cells, respectively). They obtained a comparison where the increase and morphological change of the cells over time was appreciated. In the present study, these progressive changes in cells are corroborated by the appearance of cytoplasmic prolongations (filopodia and lamellipodia) and the acquisition of a more elongated shape, as well as an increase in cell density.

Taking into account studies such as that of Ghayor et al. [18], which concluded that a low degree of microporosity and high mechanical strength yield optimal osteoconduction and creeping substitution, as well as the work of Kang [19], where the properties of compounds such as PEEK combined with HA from 10% to 30% were analyzed, highlighting the importance of the influence of particle size and dispersion uniformity to improve mechanical properties, the present study focuses on the properties of PLA combined with HA. Since the properties of hydroxyapatite have been widely described, this research is currently focused on finding polymers that complement these properties with optimal mechanical and osteoconductive behavior, as is the case of PLA, in addition to having a low degree of microporosity, as also demonstrated by He et al. [20]. However, the present study has been based on a novel 3D printed material that has a lower particle size and microporosity, so it was necessary to analyze the results obtained.

PLA is combined with HA in different concentrations for several reasons, among which are: the increase of its bioactive activity (thus increasing osteogenesis and osteoconductivity) and improvement of its mechanical properties (such as recovery stress and PLA deformation) as described by Chen et al. [21] and Al Bahrawy [22].

In the work of Zimina et al. [23] in which they compare the adhesion of pure PLA, PLA with HA 15% and PLA with HA 20%, we find adhesion levels of 6%, 8% and 19% respectively. These data seem to indicate that the higher the HA content, the cell adhesion increases, in addition to seeing a greater cell proliferation in PLA scaffolds with HA than pure PLA scaffolds. In our case, comparing the different materials with each other and with the control group, we can observe that the highest level of early cell adhesion is also found in the PLA group with 20% HA.

It should be noted that cell adhesion also depends on the surface characteristics of the material [14]. Therefore, values such as peak-valley distance, average roughness, skewness and kurtosis determine that cells adhere more easily to the material. This could explain the greater adhesion of the cells to the control group, which consisted of plastic discs without surface patterns, but specially prepared to facilitate adhesion. Therefore, an issue to be analyzed would be whether adhesion and mechanical properties improve by modifying the topographic characteristics of the scaffold (such as roughness) as investigated in the articles by Marella et al. [24], where it is noted that a rougher surface of the reduced graphene oxide/polycaprolactone scaffold increased the viability, cell propagation and differentiation. Balaji Raghavendran et al. [25] postulate that surface roughness and pore size induce cell differentiation within the scaffold and Campos-Bijit et al. [15] indicate, looking at the samples at the FESEM, that on implant surfaces with regular and moderately rough topography, cell adhesion is higher. However, this study did not use any special micro-pattern that affected the surface of the samples, but the cells adhered to the random pattern that was created by means of 3D printing. Therefore, it would be convenient to analyse the surface characterisation of these materials to observe the roughness values that can condition this adhesion.

As we have mentioned, scaffolds can be manufactured through high-precision 3D printing, since, as Frone et al. [26] point out, PLA has great fluidity and thus it can be ensured that the scaffold meets the necessary mechanical resistance, but at the same time has sufficient porosity to allow cell adhesion and recreates a precise shape of the shape of the defect [7].

Various studies have analyzed how different cells (such as PDLSCs, osteoblasts, fibroblasts, mesenchymal stem cells, etc.) adhere to different materials (including PLA, collagen, cellulose, alginate, PLC, polyethylene glycol, etc.) used in the manufacture of scaffolds [12]. In the present study, PDLSCs were chosen because studies such as those of Lee et al. [13] and Ge et al. [14] have demonstrated their efficacy in the regeneration of dental and periodontal tissues.

PLA is combined with HA in different concentrations for several reasons, including the enhancement of its bioactivity (thus promoting osteogenesis and osteoconductivity) and the improvement of its mechanical properties (such as recovery stress and deformation), as described by Chen et al. [20] and Al Bahrawy [21].

In the work of Zimina et al. [22], which compared the adhesion of pure PLA, PLA with 15% HA, and PLA with 20% HA, adhesion levels of 6%, 8% and 19% were reported, respectively. These data suggest that higher HA content promotes greater cell adhesion, along with increased cell proliferation in PLA/HA scaffolds compared with pure PLA scaffolds. In our study, comparing the different materials with each other and with the control group, the highest level of early cell adhesion was also observed in the PLA/HA scaffold with 20% HA.

It should be noted that cell adhesion also depends on the surface characteristics of the material [14]. Parameters such as peak-valley distance, average roughness, skewness, and kurtosis influence the ability of cells to adhere to the surface. This may explain the greater adhesion of cells to the control group, which consisted of plastic disks without surface patterns but specially prepared to facilitate adhesion. A relevant issue to analyze would therefore be whether adhesion and mechanical properties can be improved by modifying the topographic characteristics of the scaffold (such as roughness). For example, Marella et al. [23] reported that increased roughness in reduced graphene oxide/polycaprolactone scaffolds enhanced cell viability, proliferation, and differentiation. Similarly, Balaji Raghavendran et al. [24] postulated that surface roughness and pore size induce cell differentiation within the scaffolds, and Campos-Bijit et al. [15] observed under FESEM that cell adhesion was higher on implant surfaces with regular and moderately rough topography. However, in the present study, no special micro-patterning was applied to the samples; instead, the cells adhered to the random patterns generated by 3D printing. Therefore, future analysis of the surface characterization of these materials would be useful to determine the roughness values that may condition cell adhesion.

As previously mentioned, scaffolds can be manufactured using high-precision 3D printing, since, as noted by Frone et al. [26], PLA exhibits excellent fluidity. This property ensures that the scaffold meets the required mechanical resistance while maintaining sufficient porosity for cell adhesion and accurately reproducing the geometry of the defect [7].

The results obtained in the present study using the cell viability assay (MTT) revealed a high proportion of viable cells in all materials and concentrations, with differences observed across time points and elutions, although not all were statistically significant. When comparing the different concentrations, no statistically significant differences were observed between the control and the experimental groups, indicating that PLA and HA can be considered biocompatible materials. Similarly, no differences were detected between the materials across elutions, although PLA/HA 20% consistently demonstrated higher cell viability in most elutions. With respect to time, at 24 h, PLA/HA 20% showed significantly higher viability compared with the other materials and the control.

These findings are consistent with previous studies on the biocompatibility of PLA, such as those by Zhang [9] and Zimina [22]. In the latter, samples of pure PLA, PLA/HA 15%, and PLA/HA 20% were tested with multipotential mesenchymal stromal cells. The highest cell viability was observed in the PLA/HA 20% sample at 48 h, whereas in the present study, this peak was found across all elutions.

However, in the present study, at 48 h, the highest viability was observed in PLA/HA 10%, suggesting that over time, the effect of PLA/HA 20% diminishes. Viability tended to increase across all materials, influenced more by the passage of time than by concentration. In any case, no cytotoxic effects on PDLSCs were detected in any of the PLA/HA samples.

In the study conducted by He et al. [27], the cytotoxicity of PLA was evaluated in an osteoblastic cell line, and it was concluded that PLA exhibits excellent biocompatibility. Additional advantages were also reported when modifying scaffold components, such as improved adhesion and cell growth derived from the incorporation of chitosan into PLA, highlighting the potential for combining PLA with a wide range of compounds.

The cell viability results from the apoptosis assay in the present study revealed statistically significant differences between materials according to concentration, with PLA/HA 20% showing the highest cell survival. Regarding elutions, PLA/HA 20% also performed best. At 24 and 48 h, PLA/HA 20% exhibited significantly lower levels of necrosis and apoptosis compared with both the control and the other materials. By 72 h, however, PLA/HA 15% showed the highest percentage of viable cells, with statistically significant results. At this time point, the other concentrations also showed improved viability, although without reaching statistical significance. It follows that, over time, the samples exhibited a greater tendency toward viable cells compared with the control group.

Another study carried out by Pérez-Dávila [6] evaluated PLA with HA at different weight percentages: a control group consisting of pure PLA (without HA) and groups containing 3.9 and 13% HA by weight. Results were observed at 7, 14, and 21 days using the MTS assay and the MG-63 human osteosarcoma cell line. At 7 days, cell viability in the control group was comparable to that of the HA-containing samples. At 14 days, viability increased in all samples compared with the 7-day results, particularly in PLA with 13% HA. The same trend was observed at 21 days, although in this case, PLA with 3% HA by weight showed the most pronounced increase. The study concluded that the significant rise in viability across all samples over time confirmed the non-toxic properties of PLA/HA. Although the observation period in that study was relatively short, it suggests that, over time, cells in contact with PLA and HA adapt and proliferate, with HA concentration not a major determinant of viability.

By contrast, the study by Xu et al. [28] on C6 cells yielded results inconsistent with those above. Using the MTT assay with different concentrations of HA (0, 10, 100, 250, and 500 μg/mL) for 24, 36, and 48 h, they reported that increasing HA concentration and exposure time reduced cell viability. Similarly, in the apoptosis assay at 48 h, they observed that HA induced apoptosis in a concentration-dependent manner. The use of a different cell line and the application of high HA concentrations may explain why these findings differ from those of other studies.

Therefore, it is necessary to conduct studies with longer observation periods, assessing both pure PLA and a wider range of HA concentrations, together with additional cell lines involved in bone regeneration, to provide more robust data for comparison. It should also be noted that this study was conducted under in vitro conditions, which may influence the results due to their high sensitivity but limited specificity, as well as challenges in reproducing an adequate physiological environment, the risk of sample contamination, and restrictions related to observation time.

## 5. Conclusions

PLA combined with HA at different concentrations is a biocompatible material that supports early adhesion of periodontal ligament cells. No significant differences were observed between PLA/HA concentrations or between PLA/HA and the control disks; however, PLA/HA 20% generally demonstrated superior results in terms of adhesion levels and reduced apoptosis or cell death. The cells developed extensions of the cytoplasm to facilitate the connection between cells with each other faster in PLA/HA 20%. Over time, proliferation, adhesion, and viability of PDLSCs increased across all PLA/HA concentrations.

## Figures and Tables

**Table 1:** Differences between materials at 48 h.

*p* Value	PLA/HA 10%	PLA/HA 15%	PLA/HA 20%
PLA/HA 15%	**0.0001**		
PLA/HA 20%	0.6130	**0.0001**	
Control group	**0.0287**	**0.0001**	0.5593

**Table 2:** Differences between materials at 72 h.

*p* Value	PLA/HA 10%	PLA/HA 15%	PLA/HA 20%
PLA/HA 15%	0.0012		
PLA/HA 20%	0.9691	0.0133	
Control group	1.0000	0.0017	1.0000

**Table 3:** Topography and wetting mean values and SD.

Group	Topography				Wetting
	Ra (µm)	Rq (µm)	Rsk (-)	Kurtosis (-)	Contact Angle (°)
PLA/HA 10 PLA/HA 15 PLA/HA 20	2.1 (0.6) ^a^ 1.7 (0.1) ^c^ 1.1 (0.2) ^b^	2.6 (0.8) ^a^ 2.1 (0.3) ^a^ 1.4 (0.3) ^b^	−0.2 (1) ^a^ −0.1 (0.9) ^a^ 0.4 (0.9) ^a^	6 (2) ^a^ 3 (1) ^b^ 7 (3) ^a^	40 (2) ^a^ 48 (3) ^c^ 30 (2) ^b^

SD: Standard deviation; Values in each column with different letter are statistically different (*p* < 0.05); Ra: mean roughness; Rq: squared roughness; Rsk: Skewness; Rk: Kurtosis.

## Data Availability

The original contributions presented in this study are included in the article. Further inquiries can be directed to the corresponding author.

## Abbreviations

The following abbreviations are used in this manuscript:

PLDSC	Periodontal ligament stem cells
HAS	Hydroxyapatite
PLA	Polylactic acid
MTT	3-(4,5-dimethylthiazol-2-yl)-2,5-diphenyltetrazolium bromide
DMEM	Dulbecco modified Eagle medium
DMSO	Dimethyl sulfoxide
